# Cohorting Dengue Patients Improves the Quality of Care and Clinical Outcome

**DOI:** 10.1371/journal.pntd.0003836

**Published:** 2015-06-05

**Authors:** Lucy C. S. Lum, Sharifah Faridah Syed Omar, Sasheela Sri La Sri Ponnampalavanar, Lian H. Tan, Shamala Devi Sekaran, Adeeba Kamarulzaman

**Affiliations:** 1 Department of Paediatrics, Faculty of Medicine, University of Malaya, Kuala Lumpur, Malaysia; 2 Department of Medicine, Faculty of Medicine, University of Malaya, Kuala Lumpur, Malaysia; 3 Department of Medicine, Faculty of Medicine, University of Malaya, Kuala Lumpur, Malaysia; 4 Department of Medicine, Sunway Medical Centre, Petaling Jaya, Selangor, Malaysia; 5 Department of Medical Microbiology, Faculty of Medicine, University of Malaya, Kuala Lumpur, Malaysia; 6 Department of Medicine, Faculty of Medicine, University of Malaya, Kuala Lumpur, Malaysia; University of Rhode Island, UNITED STATES

## Abstract

**Introduction:**

The increasing incidence of dengue among adults in Malaysia and other countries has important implications for health services. Before 2004, in order to cope with the surge in adult dengue admissions, each of the six medical wards in a university hospital took turns daily to admit and manage patients with dengue. Despite regular in-house training, the implementation of the WHO 1997 dengue case management guidelines by the multiple medical teams was piecemeal and resulted in high variability of care. A restructuring of adult dengue inpatient service in 2004 resulted in all patients being admitted to one ward under the care of the infectious disease unit. Hospital and Intensive Care Unit admission criteria, discharge criteria and clinical laboratory testing were maintained unchanged throughout the study period.

**Objectives:**

To evaluate the impact of cohorting adult dengue patients on the quality of care and the clinical outcome in a university hospital in Malaysia.

**Methods:**

A pre (2003) and post-intervention (2005–6) retrospective study was undertaken.

**Intervention:**

Cohorting all dengue patients under the care of the Infectious Disease team in a designated ward in 2004.

**Results:**

The number of patients enrolled was 352 in 2003, 785 in 2005 and 1158 in 2006. The evaluation and detection of haemorrhage remained high (>90%) and unchanged throughout the study period. The evaluation of plasma leakage increased from 35.4% pre-intervention to 78.8% post-intervention (p = <0.001) while its detection increased from 11.4% to 41.6% (p = <0.001). Examination for peripheral perfusion was undertaken in only 13.1% of patients pre-intervention, with a significant increase post-intervention, 18.6% and 34.2% respectively, p = <0.001. Pre-intervention, more patients had hypotension (21.5%) than detected peripheral hypoperfusion (11.4%), indicating that clinicians recognised shock only when patients developed hypotension. In contrast, post-intervention, clinicians recognised peripheral hypoperfusion as an early sign of shock. The highest haematocrit was significantly higher post-intervention but the lowest total white cell counts and platelet counts remained unchanged. A significant and progressive reduction in the use of platelet transfusions occurred, from 21.7% pre-intervention to 14.6% in 2005 and 5.2% in 2006 post-intervention, p<0.001. Likewise, the use of plasma transfusion decreased significantly from 6.1% pre-intervention to 4.0% and 1.6% in the post-intervention years of 2005 and 2006 respectively, p<0.001. The duration of intravenous fluid therapy decreased from 3 days pre-intervention to 2.5 days (p<0.001) post-intervention; the length of hospital stay reduced from 4 days pre- to 3 days (p<0.001) post-intervention and the rate of intensive care admission from 5.8% pre to 2.6% and 2.5% post-intervention, p = 0.005.

**Conclusion:**

Cohorting adult dengue patients under a dedicated and trained team of doctors and nurses led to a substantial improvement in quality of care and clinical outcome.

## Author Summary

The epidemiology of dengue disease in the tropical regions is characterized by a rapid increase in the number of reported cases, and in some Asian countries, a shift in the age range predominance from children toward adults. This has important implications for health services, resources, and training, provision of treatment and care and ultimately clinical outcome of dengue. There is no literature on the impact of this shift in the epidemiological pattern of dengue on healthcare delivery and the clinical outcome of adult dengue patients. Before 2004, each of the 6 general medical wards in a university hospital took turns daily to admit and manage patients with dengue. Despite regular in-house training, the implementation of the WHO 1997 dengue case management guidelines by the multiple teams was piecemeal with high variability in clinical practice. In 2004 all dengue patients were admitted to one ward under the care of the infectious disease unit. In this pre (2003) and post-intervention (2005–6) retrospective study we demonstrated that cohorting dengue patients under a specific team enhances the experiential knowledge of clinicians in managing dengue patients from the perspective of clinical evaluation and detection of increased vascular permeability and the ensuing hypovolemic shock with improvement in clinical outcomes.

## Introduction

Dengue is the most rapidly advancing vector-borne disease of public health significance in the tropics. Before 1970, only nine countries had experienced severe dengue outbreaks. In the last 50 years, the incidence of dengue has increased 30 fold; more than 2.5 billion people (>40% of world’s population) live in >100 endemic countries [1].

Its more severe form, dengue haemorrhagic fever (DHF) was typically a childhood disease in Southeast Asia with deaths occurring mainly in children. Since the 1980’s, however there has been an increasing incidence of DHF among older age groups in Latin America and Southeast Asia [2].

The epidemiology of dengue disease in Malaysia was characterized by a non-linear increase in the number of reported cases from 7,103 in 2000 to 46,171 in 2010, and a shift in the age range predominance from children toward adults [3]. The proportion of dengue-related deaths aged > 15 years similarly increased from 49% of total dengue deaths in 1999, to 86% in 2006 [4]. The phenomenon of predominantly adult dengue population has also been observed in Singapore and Taiwan [5–8]. Other countries that have reported increased dengue infection in adults include Thailand [9–10], India [11], Bangladesh [12], Cuba [13–14] and Brazil [15].

The transition in the epidemiology of dengue from a childhood disease to one that affects the older age group was reflected in hospital admissions throughout urban areas of Malaysia. The shift in the modal age group of DHF from children to the adult population has important implications for health services, resources, and training, provision of treatment and care and ultimately clinical outcome of dengue. To date there is no literature on the impact of this shift in the epidemiological pattern of dengue on healthcare delivery and the clinical outcome of adult dengue patients.

University of Malaya Medical Centre (UMMC) is a teaching hospital situated in the Klang Valley that has recorded a large number of dengue related outpatient attendance and hospital admissions over the last several years. Patients with suspected dengue who required inpatient care were admitted through the Department of Primary Care Medicine or through the Emergency Department to the Departments of Paediatrics or Medicine respectively. Before 2004, in order to cope with the surge in adult dengue admissions, each of the 6 general medical wards in UMMC took turns daily to admit and manage patients with dengue. Within each ward, care was delivered by a medical team comprising of the respective consultant, specialists, and several registrars and interns who rotated through the general medical wards. Despite regular in-house training, the implementation of the WHO 1997 dengue case management guidelines [16] by the multiple medical teams was piecemeal and resulted in high variability of care which included the transfusion of blood products. This situation prompted a restructuring of adult dengue inpatient service in 2004, resulting in all these patients being admitted to one ward under the care of the infectious disease (ID) unit, led by an ID consultant, a specialist and 2 to 3 registrars and rotating interns. On-going training in dengue case management was continued as before. Critically ill dengue patients were admitted to the Intensive Care Unit (ICU) under the care of Department of Anaesthesiology and Intensive Care. Hospital and ICU admission criteria, discharge criteria and clinical laboratory testing were maintained unchanged throughout the study period. The objective of our study was to evaluate the impact of cohorting of dengue inpatients under the care of the ID unit, on the quality of care and the clinical outcome in adult dengue patients in UMMC.

## Methods

This is a retrospective study that looked at a registry of inpatients admitted to adult medical wards with a discharge diagnosis of dengue in 2003 (pre-intervention), 2005 and 2006 (post-intervention). Relevant records were obtained from the Medical Records Department in a chronological order. Patients admitted in 2004, the year of implementation of the policy, were excluded from the study. Due to the inability to retrieve all relevant past medical records, the targeted percentage of patients randomly selected to be included in the study was at least 50%, 70% and 80% for admissions in 2003, 2005 and 2006 respectively. When the medical record of a randomly selected patient could not be traced, the patient up on the unselected list was enrolled. Data was abstracted into a standardised case report form by 3 dedicated trained nurses. Data included demographics, clinical features pertinent to dengue management which included any evaluation of haemorrhage, plasma leakage and shock. Treatment parameters included duration of intravenous fluid therapy, use of platelet transfusions and other blood products and length of hospital stay. The study was approved by the Ethics Committee of UMMC.

The presence or absence of any haemorrhage including skin or mucosal bleeding, either spontaneous or induced such as bruises following venepunctures were recorded. Gastrointestinal (GI) bleeding referred to either hematemesis or malena. Documentation of evidence of plasma leakage including clinical or radiological detection of any of the following: pleural effusion, or ascites; or haemoconcentration as defined by 20% or more increase in highest haematocrit above that at discharge. Assessment of peripheral perfusion included any of the following: capillary refill time, temperature and colour of extremities and volume of a peripheral pulse. Peripheral hypoperfusion was recorded as being present when any of the parameters of peripheral perfusion were abnormal. Hypotension was defined as systolic blood pressure below 90 mmHg for males and 85 mmHg for females and narrowed pulse pressure was when it was < 20 mmHg. Duration of intravenous fluid (IVF) therapy was rounded off to the nearest 0.5 day.

### Data Analysis

All data were analysed using SPSS for Windows, version 20. Data from the post-intervention group (2005 and 2006) was compared with that of the pre-intervention group (2003). The Chi-square and Mann-Whitney tests were used where appropriate. The level of statistical significance for all analyses was set at *p*<0.05 using 2-tailed comparisons.

## Results

Results of the study are shown in Tables 1 and 2. During the study period, the number of adult dengue inpatients increased progressively from 671 in 2003 to 1096 and 1,404 in 2005 and 2006 respectively. The number of patients enrolled into the study was 352 (52.4% of admissions), 785 (71.6%) and 1158 (82.5%) in 2003, 2005, 2006 respectively. The slight predominance of male patients increased significantly from pre-intervention to post-intervention period and this pattern was also observed in the corresponding total dengue inpatients. There was no difference in the median age of the enrolled population and the median duration of illness at the time of admission throughout the study period.

**Table 1:** Summary of results: Demographics and dengue IgM serology status.

Year	2003 (Pre-intervention)	2005 + 2006 (Post-intervention)	P value
Total adult dengue admissions	671	2500	
Male	356 (53)	1440 (57.6)	
Mortality	5 (0.75)	14 (0.56)	0.857
**Demographics**			
Enrolled subjects	352 (52.4)	1943 (77.7)	<0.001
Male	179 (50.9%)	1141 (58.7)	0.004
Age	27.7 (21.6–37.5)	26.6 (21.4–35.0)	0.596
Duration of illness on admission (day)	5 (5–6)	6 (5–6)	0.454
**Dengue serology (igM)**			
Serological Positive	232 (69.5)	1805 (92.9)	<0.001
2 or more samples sent for IgM	41 (11.9)	1102 (56.7)	<0.001

**Table 2:** Summary of results: Clinical parameters, laboratory parameters, treatment and outcome.

Year	2003 (Pre-intervention) n = 352	2005 + 2006 (Post-intervention) n = 1943	P value
**Clinical Parameters**			
Available data on any haemorrhage	349 (99.1)	1931 (99.4)	0.875
Presence of any haemorrhage	202 (57.4)	1111 (57.2)	0.875
GI Bleed	16 (4.6)	112 (5.8)	0.523
Available data on plasma leakage	126 (35.8)	1393 (71.7)	<0.001
Presence of plasma leakage	40 (11.4)	650 (34.4)	<0.001
Available data on peripheral perfusion	46 (13.1)	537 (27.6)	<0.001
Presence of hypoperfusion	40 (11.4)	457 (23.5)	<0.001
Hypotension or narrowed pulse pressure	75 (21.5)	190 (9.8)	<0.001
Ratio of hypoperfusion to hypotension	0.53	2.41	
**Laboratory Parameters**			
Highest HCT before IVF (%)	43 (40–47)	45.7 (42–49)	<0.001
Highest HCT throughout admission (%)	44 (41–48.75)	46.0 (42–50)	0.001
Lowest WBC (x10^9^/L)	2.8 (2.0–3.9)	2.7 (1.9–3.7)	0.183
Lowest platelet count (x10^9^/L)	31 (18–53)	30 (16–47)	0.672
Lowest platelet count of transfused (x10^9^/L)	13 (9–19)	9.5 (6–17)	<0.001
Lowest platelet count of not transfused (x10^9^/L)	40 (25–57)	32 (19–50)	0.001
**Treatment**			
Days on IVF	3 (2–4)	2.5 (2–3.0)	<0.001
Platelet transfusion	75 (21.7)	174 (9.0)	<0.001
Plasma transfusion	21 (6.1)	50 (2.6)	0.003
Blood transfusion	6 (1.7)	21 (1.1)	0.555
Use of antibiotics	44 (12.5)	180 (9.3)	0.046
**Outcome**			
LOS (days)	4 (3.0–5.0)	3 (3.0–4.0)	<0.001
Intensive Care Unit Admission	20 (5.8)	49 (2.5)	0.002

Data is expressed as number (%) or median (interquartile range). GI is gastrointestinal, HCT is haematocrit, WBC is total white cell count, IVF is intravenous fluid, LOS is Length of stay.

The documentation of haemorrhagic manifestations by physicians was consistently high (<90%) during the pre and post-intervention periods, with no significant difference in the observed incidence of any bleeding (57%) or the more serious gastro-intestinal bleeding (4.5% to 5.8%) throughout the study period. In contrast, only 35.8% of patients’ medical records had any documentation of plasma leakage during the pre-intervention period; but any documentation of this phenomenon by doctors increased significantly and progressively during the 2 years post-intervention to 71.7%, p<0.001, being 59% and 78.8% in 2004 and 2005 respectively. Together with increased detection for plasma leakage, so its presence increased, from 11.4% pre-intervention to 34.4%, post-intervention, p<0.001, (13,8% in 2005 and 47.6 in 2006). Likewise, examination for peripheral perfusion was undertaken in only 13.1% of patients pre-intervention, with a significant increase to 27.6% post-intervention, p = <0.001 (18.6% and 34.3% respectively). The presence of peripheral hypoperfusion increased significantly together with an increase in documentation of this physical sign, from 11.4% pre-intervention to 23.5% post-intervention, p<0.001, (14.1% and 29.9% respectively). Conversely, the incidence of hypotension and narrowed pulse pressure decreased significantly post-intervention, 9.8%, from 21.5% pre-intervention, p<0.001, (10.2% and 9.5% respectively). The ratio of patients observed to have peripheral signs of hypoperfusion to those with hypotension increased from 0.53 pre-intervention to 2.41, post-intervention. The highest haematocrit (HCT) and the HCT before the start of intravenous fluid was significantly higher post-intervention. There was no significant difference in the lowest total white cell count and lowest platelet count throughout the illness during the pre and post-intervention periods.

The duration of intravenous fluid therapy showed a slight but significant decrease from a median of 3 days pre-intervention to a median of 2.5 days (p<0.001) post-intervention. Similarly, the length of hospital stay, reduced from a median of 4 days pre- to that of 3 days (p<0.001) post-intervention. In addition, a significant reduction in the use of platelet transfusions was observed, from 21.7% pre-intervention to 9% post-intervention, p<0.001 (14.6% in 2005 and 5.2% in 2006). Likewise, the use of plasma transfusion decreased significantly from 6.1% pre-intervention to 2.6% post-intervention, p = 0.003 (4.0% and 1.6% in 2005 and 2006 respectively). Although there was no difference in the lowest platelet counts there was, however, a significant difference in the lowest platelet count among patients who received platelet transfusions, 13 x10^9^/L pre-intervention to 9.5% post-intervention (10 x10^9^/L and 8 x10^9^/L in 2005 and 2006 respectively), p<0.001. The lowest platelet counts in patients who did not receive platelet transfusions decreased significantly during the post-intervention, p = 0.001. There was a decrease in use of blood transfusion and antibiotics during the post-intervention period, the latter being a significant reduction, p = 0.046. The rate of ICU admissions decreased significantly from 5.8% pre to 2.5% post-intervention, p = 0.002. The overall mortality rate decreased from 0.75% pre-intervention to 0.56% post-intervention, this difference was however not significant, p = 0.857. The rate of positive IgM for dengue increased significantly post-intervention, from 69.5% pre to 93% post intervention, p<0.001, with significantly more patients, 34.3% and 56.7% respectively having repeated serology tests, p<0.001.

## Discussion

Our study demonstrates that cohorting dengue patients under the care of a trained team of clinicians were associated with several changes in clinical practice. The pre and post-intervention groups of patients were comparable in their median age and the day of illness at the time of admission. They were comparable in the severity of illness as represented by the presence of any haemorrhage, gastrointestinal haemorrhage, and the lowest platelet and white blood cell counts.

While the majority of clinicians were well aware of the haemorrhagic manifestations of dengue, only a minority were knowledgeable of plasma leakage before the intervention. The higher level of awareness of clinicians to haemorrhagic manifestations may be due to the term “dengue haemorrhagic fever” which placed emphasis on haemorrhage rather than plasma leakage as the main pathophysiology of severe dengue. Under-recognition of plasma leakage was identified as one of the difficulties causing the inappropriate classification of dengue using the 1997 WHO dengue case classification [17, 18]. With repeated exposure over a period of time, a progressive increase in awareness among clinicians to the unique feature of plasma leakage and the ensuing shock led to a practice where these physical signs were actively elicited. Clinical detection for plasma leakage increased from 2003 to 2006 resulting in a higher number of patients identified with this complication. A similar but smaller increase in clinicians’ awareness to peripheral signs of shock was also noted. As a result of an increase in the detection of peripheral hypoperfusion, which precedes hypotension, the incidence of central hypoperfusion among dengue inpatients decreased. More patients were detected to have hypotension (21.5%) than hypoperfusion (11.4%) pre-intervention, indicating that clinicians recognised shock only when patients developed hypotension, a finding similar to that of an earlier study in 2002 [17]. In contrast, during the post-intervention period, clinicians recognised peripheral hypoperfusion as an early sign of shock. A slight but significant increase in the highest haematocrit from the pre-intervention to post-intervention period suggested that the latter patients could have more severe plasma leakage. It is possible that a few hours of delay in blood sampling during the plasma leakage phase of illness, might result in an increase in haematocrit. This delay might be due to a larger volume of inpatients during the post-intervention phase. However, earlier detection and correction of hypovolaemia might have prevented patients from progressing to hypotensive shock necessitating ICU management, resulting in a significant decrease in ICU admission post-intervention. There was a non-significant reduction on overall mortality observed in the post intervention compared to the pre-intervention period.

A marked decrease in the use of platelet and plasma transfusions was one of the most noticeable effects of cohorting dengue patients. During the study period, the threshold to transfuse platelets for thrombocytopenia increased significantly, from 13 x10^9^/L pre-intervention compared to 10 x10^9^/L and then 8 x10^9^/L during the post-intervention years of 2005 and 2006 respectively. Over time, experiential knowledge of thrombocytopenia being not associated with bleeding in dengue [19–21] led clinicians to develop confidence to gradually abandon the practice of platelet or plasma transfusions. The duration of administration of intravenous fluid was reduced which was associated with a significant decrease in length of hospital stay post-intervention.

Experience plays a central role in the learning process whereby knowledge and concepts are derived from and continuously modified by experience [22]. Clinical experience, based on personal observation, reflection, and judgment is needed to translate scientific results into treatment of individual patients [23]. Personal experience is often a more powerful persuader than scientific publication in changing clinical practice [24, 25]. Cohorting dengue patients under a trained team provide an environment that favours the experiential learning process. Theoretical knowledge, for example, of plasma leakage and its complications and clinical skills in detection could be continually validated and internalized through repetitive clinical exposure. This is shown in the incremental improvement of clinical practices and clinical outcomes over time. Other benefits of cohorting dengue patients include transfer of experiential knowledge from paediatricians to adult physicians, focused training of nursing staff which, in turn, contributed to better monitoring of dengue patients for symptoms and signs of plasma leakage and shock and oral and intravenous fluid balance. Teams dedicated to the case management of dengue have been successfully formed in countries such as Thailand and Viet Nam [26–28], where case fatality rates for children with DHF have been the lowest in the region.

Our study has several limitations. It was a retrospective study entirely dependent on the availability of medical records of randomly selected patients. This might have an effect on the enrolment of our patients and resulting in a selection bias. The failure to record clinical information reflected either a lack of awareness of clinicians or incomplete documentation. The fact that a high proportion of patients with documented data on haemorrhage throughout the study period suggests a high level of awareness of bleeding as a complication of dengue. Thus, a lack of documentation of evidence of plasma leakage or shock suggests a lack of awareness of these complications.

Not all dengue inpatients had laboratory confirmation of dengue, perhaps again reflecting the low level of knowledge pre-intervention, that a negative IgM for dengue will need to be repeated. The rate of seropositivity for dengue IgM increased when repeated later in the disease. It could be that changes in practices between 2003 and 2005–6 reflect overall temporal trends rather than an effect of cohorting patients, as there were progressive changes over the two years post-intervention. However, a study conducted in 2002 in the same institution and in the same setting of dispersed placement of adult dengue inpatients showed that only 7% of adult dengue inpatients with thrombocytopenia and evidence of plasma leakage were correctly classified as DHF, thus reflecting a low level of awareness among clinicians of the main pathophysiology of severe dengue [17].

Improving dengue case management is a significant priority in ensuring improved clinical outcomes. Cohorting dengue inpatients under the care of a dedicated and trained team of doctors and nurses enhanced the acquisition of experiential knowledge and clinical experience, which is translated into improved clinical practice and clinical outcome. The changes in clinical practices during the post-intervention period were the higher rates of elicitation of evidence of plasma leakage and peripheral hypoperfusion and repeat of dengue serology when negative. The observed changes in clinical outcomes were a significant reduction in duration of intravenous fluid therapy, incidence of ICU admissions and transfusions of platelets and plasma. Our study also provides evidence that preventive transfusions of blood products, widely practised in countries endemic in dengue, are unnecessary [29, 30]. By diverting the focus of management away from preventive transfusions, more attention could be given to the major problem in severe dengue, increased capillary permeability. All these would translate into substantial reduction in costs of treatment. In addition to training a dedicated team, allocating a designated ward for dengue patients in endemic countries or during a dengue outbreak should be considered as one of the effective strategies in improving clinical outcome.

## Supporting Information

S1 Checklist STROBE checklist. (DOC)

## References

[1] Dengue and severe dengue. World Health Organization [Internet]. 2015 May. http://www.who.int/mediacentre/factsheets/fs117/en/

[2] Guha-Sapir D, Schimmer B. Dengue fever: new paradigms for a changing epidemiology. Emerg Themes Epidemiol. 2005 3 2;2(1):1 15743532 10.1186/1742-7622-2-1 PMC555563

[3] Mohd-Zaki AH, Brett J, Ismail E, L’Azou M. Epidemiology of Dengue Disease in Malaysia (2000–2012): A Systematic Literature Review. PLOS Neglected Tropical Diseases. 2014 Nov, e3159. 10.1371/journal.pntd.0003159 PMC4222702 25375211

[4] Ministry of Health Malaysia, Academy of Medicine Malaysia. Clinical Practice Guidelines, Management of Dengue Infection in Adults (Revised 2^nd^ Edition) 2010.

[5] Goh KT. Changing epidemiology of dengue in Singapore. Lancet. 1995 10 21;346(8982):1098 7564804 10.1016/s0140-6736(95)91771-3

[6] Ooi EE, Goh KT, Gubler DJ. Dengue prevention and 35 years of vector control in Singapore, Emerg Infect Dis. 2006 6;12(6):887–93. 16707042 10.3201/10.3201/eid1206.051210 PMC3373041

[7] Lin CC, Huang YH, Shu PY, Wu HS, Lin YS, Yeh TM, Liu HS, Liu CC, Lei HY. Characteristic of dengue disease in Taiwan: 2002–2007. Am J Trop Med Hyg. 2010 4;82(4):731–9. 10.4269/ajtmh.2010.09-0549 20348527 PMC2844571

[8] Wang CC, Lee IK, Su MC, Lin HI, Huang YC, Liu SF, Wu CC, Lin MC. Differences in clinical and laboratory characteristics and disease severity between children and adults with dengue virus infection in Taiwan, 2002. Trans R Soc Trop Med Hyg. 2009 9;103(9):871–7. 10.1016/j.trstmh.2009.04.024 19500813

[9] Chareonsook O, Foy HM, Teeraratkul A, Silarug N. Changing epidemiology of dengue hemorrhagic fever in Thailand. Changing epidemiology of dengue hemorrhagic fever in Thailand Epidemiol Infect. 1999 2;122(1):161–6. 10098800 10.1017/s0950268898001617 PMC2809602

[10] Buathong R, Iamsiritaworn S, Siriarayapon P, Panket P, Anantapreecha S, O’Reilly M, Ungchusak K. Changing Epidemiology of Dengue Infection in Thailand: Evidence from an Outbreak Investigation in Rural Villages of Sukhothai Province, Thailand, 2006. Weekly Epidemiological Surveillance Report Vol. 44 Supplement: March 2013. S8-14. http://www.boe.moph.go.th/

[11] Gupta E, Dar L, Kapoor G, Broor S. The changing epidemiology of dengue in Delhi, India. Virol J. 2006 11 5;3:92 17083743 10.1186/1743-422X-3-92 PMC1636631

[12] Rahman M, Rahman K, Siddque AK, Shoma S, Kamal AH, Ali KS, Nisaluk A, Breiman RF. First outbreak of dengue hemorrhagic fever, Bangladesh. Emerg Infect Dis. 2002 7;8(7):738–40. 12095447 10.3201/eid0807.010398 PMC2730336

[13] Kouri GP, Guzmán MG, Bravo JR, Triana C. Dengue haemorrhagic fever/dengue shock syndrome: lessons from the Cuban epidemic, 1981. Bull World Health Organ. 1989;67(4):375–80. 2805215 PMC2491263

[14] Guzmán MG1, Kouri G, Valdes L, Bravo J, Alvarez M, Vazques S, Delgado I, Halstead SB. Epidemiologic studies on Dengue in Santiago de Cuba, 1997. Am J Epidemiol. 2000 11 1;152(9):793–9; discussion 804. 11085389 10.1093/aje/152.9.793

[15] Guilarde AO, Turchi MD, Siqueira JB Jr, Feres VC, Rocha B, Levi JE, Souza VA, Boas LS, Pannuti CS, Martelli CM. Dengue and dengue hemorrhagic fever among adults: clinical outcomes related to viremia, serotypes, and antibody response. J Infect Dis. 2008 3 15;197(6):817–24. 10.1086/528805 18269315

[16] World Health Organisation. Dengue haemorrhagic fever: diagnosis, treatment, prevention and control 2nd edition Geneva: WHO 1997.

[17] Ng CF, Lum LC, Ismail NA, Tan LH, Tan CP. Clinicians' diagnostic practice of dengue infections. J Clin Virol. 2007 11;40(3):202–6. 17928264 10.1016/j.jcv.2007.08.017

[18] Bandyopadhyay S, Lum LC, Kroeger A. Classifying dengue: a review of the difficulties in using the WHO case classification for dengue haemorrhagic fever. Trop Med Int Health. 2006 8; 11(8):1238–55. 16903887 10.1111/j.1365-3156.2006.01678.x

[19] Chuansumrit A, Phimolthares V, Tardtong P, Tapaneya-Olarn C, Tapaneya-Olarn W, Kowsathit P, et al Transfusion requirements in patients with dengue hemorrhagic fever. Southeast Asian J Trop Med Public Health. 2000;31:10–14. 11023057

[20] Lum LC, Abdel-Latif Mel-A, Goh AY, Chan PW, Lam SK. Preventive transfusion in Dengue shock syndrome-is it necessary? J Pediatr. 2003 11;143 (5):682–4. 14615749 10.1067/s0022-3476(03)00503-1

[21] Chaudhary R, Khetan D, Sinha S, Sinha P, Sonker A, Pandey P, Das SS, Agarwal P, Ray V. Transfusion support to Dengue patients in a hospital based blood transfusion service in north India. Transfus Apher Sci. 2006 12;35(3):239–44. 17097349 10.1016/j.transci.2006.08.007

[22] Kolb DA. 1984 Experiential learning: Experience as the source of learning and development Englewood Cliffs, NJ: Prentice-Hall http://academic.regis.edu/ed205/kolb.pdf (31.05.2006).

[23] Freidson E. Profession of medicine: a study of the sociology of applied knowledge New York: Dodd, Mead and Company; 1970.

[24] Kanouse DE, Jacoby I. When does information change practitioners' behavior? Int J Technol Assess Health Care. 1988;4(1):27–33. 10287110 10.1017/s0266462300003214

[25] P Burdett-Smith P, A patient who changed my practice: Always check the respiratory rate. BMJ 1997;314:l

[26] Kalayanarooj S. Standardized clinical management: evidence of reduction of dengue haemorrhagic fever case-fatality rate in Thailand. Dengue Bull. 1999;23:10–17.

[27] Hung NT, Lan NT. Improvement of case-management–a key factor to reduce case–fatality rate of dengue haemorrhagic fever in Southern Viet Nam. Dengue Bull. 2003;27:144–8.

[28] Mayurasakorn S, Suttipun N. The impact of a program for strengthening dengue hemorrhagic fever case management on the clinical outcome of dengue hemorrhagic fever patients. Southeast Asian J Trop Med Public Health. 2010 7;41(4):858–63. 21073059

[29] Lye DC, Lee VJ, Sun Y, Leo YS. Lack of efficacy of prophylactic platelet transfusion for severe thrombocytopenia in adults with acute uncomplicated dengue infection. Clin Infect Dis. 2009;48:1262–1265. 10.1086/597773 19292665

[30] Khan Assir MZ, Kamran U, Ahmad H, Bashir S, Mansoor H, Anees SB, Akram J. Effectiveness of platelet transfusion in dengue Fever: a randomized controlled trial. Transfus Med Hemother. 2013 10;40(5):362–8. Epub 2013 Sep 11. 10.1159/000354837 24273491 PMC3822277

